# Psychometric Properties of the Urdu Version of Diabetes Knowledge Questionnaire

**DOI:** 10.3389/fpubh.2017.00139

**Published:** 2017-06-27

**Authors:** Allah Bukhsh, Shaun Wen Huey Lee, Priyia Pusparajah, Amer Hayat Khan, Tahir Mehmood Khan

**Affiliations:** ^1^School of Pharmacy, Monash University, Bandar Sunway, Malaysia; ^2^Jeffrey Cheah School of Medicine and Health Sciences, Monash University, Bandar Sunway, Malaysia; ^3^School of Pharmaceutical Sciences, Universiti Sains Malaysia, Gelugor, Penang, Malaysia

**Keywords:** diabetes, patient knowledge, psychometric analysis, HbA1c, Pakistan

## Abstract

**Objective:**

Patient education is a key element in the treatment of diabetes. Assessment of diabetes knowledge is important for optimum treatment. For the assessment of diabetes knowledge, validated tool is essential. None of such validated tool is available in Urdu language. Therefore, the aim of this study was to translate and examine the psychometric properties of the 24-item Urdu version of Diabetes Knowledge Questionnaire (DKQ) among type 2 diabetes patients.

**Methods:**

Standard “forward–backward” process was used to translate DKQ into Urdu language. Later, it was validated on a convenience sample of 130 patients with type 2 diabetes, between July and September 2016. Internal consistency was assessed by reliability analysis, one-way analysis of variance was applied for known group validity and multivariate linear logistic regression was applied for identifying significant predictors for patients’ DKQ score.

**Results:**

Good internal consistency was observed for DKQ (Cronbach’s α = 0.702). The mean HbA1c of the patients was 8.55% (±1.91). DKQ scores of patients’ with “good glycemic control” (14.22 ± 2.4) were observed significantly higher (*P* < 0.05) than patients with “poor glycemic control” (12.56 ± 2.75). Multiple linear regression revealed that patients’ HbA1c (OR −0.17, CI −1.111, −0.023) and patients’ education (OR 0.17, CI −0.032, 0.758) were significant predictors for DKQ sum score.

**Conclusion:**

Urdu version of the DKQ is a valid and reliable instrument for adequate estimation of disease knowledge and its association with glycemic control in type 2 diabetes patients in Pakistan.

## Introduction

Diabetes, one of the most common non-communicable disease worldwide (1). According to International Diabetes Federation, approximately 415 million people living with diabetes mellitus (DM) worldwide, which is expected to reach 642 million by 2040 (2). Pakistan has been ranked 7th in diabetes disease burden in the world with prevalence rate of 7.6–11% in 2011, it is projected to reach 15% (14 million) by 2030. If the present scenario continues, Pakistan is expected to move to top 4th place (3).

Type 2 diabetes mellitus (T2DM) is a metabolic disorder and its optimum management requires not only medication use but an adequate patient disease knowledge and self-care behaviors. Poor diabetes control lead toward increased risk of micro- and macro-vascular complications, e.g., diabetic nephropathy, diabetic neuropathy, coronary arty disease, and diabetic foot (4). Diabetes is a lifelong disorder and comorbidities associated with its poor control impose an enormous economic burden on individual, society, and health-care system (5, 6). However, effective glycemic control may impede the chances of such diabetes associated morbidities and mortalities (7).

Glycemic control is one of the major goals of diabetes management. Glycemic control can be evaluated with the help of glycated hemoglobin (HbA1c), which represents average blood glucose levels of previous 2–3 months period. For this reason, HbA1c is known to be the best indicator for long-term glycemic control in people with DM (7). In order to achieve good glycemic control, it is necessary to measure glycated hemoglobin (HbA1c) and assess patient’s diabetes knowledge.

Although diabetes disease knowledge alone does not ensure the desired modifications for effective self-care, yet diabetes knowledge is an important first step to measure the outcome and optimization of the patient education programs (8–10). Nevertheless, easy to administer, reliable, and valid tools to patients’ diabetes knowledge are scarce (9). It is the need of time to run effective diabetes educational awareness programs for people to educate them about their life style interventions (11, 12). This will greatly help to decrease diabetes prevalence.

To assess the patients’ diabetes knowledge, Diabetes Knowledge Questionnaire (DKQ) has been developed by the Starr County Diabetes Education Study (10). DKQ was developed in English language and has been translated and validated in many languages, but it has not been translated and validated in Pakistan. Therefore, study aim was to translate and validate DKQ among T2DM patients in Urdu language in Pakistan, as Urdu is the national language of Pakistan and is widely spoken by its population. Additionally, the degree of association of diabetes knowledge will be investigated with glycemic control (HbA1c) in the present study.

## Materials and Methods

### Study Design

Cross-sectional study design was adopted for data collection. A target convenience sample of 160 patients with type 2 DM was estimated. This validation study was performed from July to September 2016 at Akhuwat Hospital Lahore, Pakistan, and Awan Medical Complex Lahore, Pakistan.

### Instrument Translation

From the many available published questionnaires, we selected the Diabetes Knowledge Questionnaire 24, developed for the Starr County Diabetes Education Study. This tool originally developed with 60 items was later abridged to 24 items. Permission of the original authors was obtained for translation and usage of DKQ. The questionnaire was translated into Urdu using a standardized forward and backward translation procedure, as recommended by Bradley (13), briefly, the translation process involved two native linguistic experts who translated word to word from the original English DKQ version to Urdu version (forward translation). For rechecking the adequacy of the translation, the translated questionnaire was submitted to the linguistic department, who proposed some minor grammatical corrections. Upon correcting these errors, forward translation phase of the tool was completed. Later on, two linguistic experts translated back the corrected Urdu version to English version (backward translation), the resulting discrepancies were resolved resulting in the finalized Urdu version for face validity testing. The translated DKQ Urdu version was administered to 20 Urdu speaking diabetes patients, attending outpatient department of the hospital for face validity followed by appropriate modifications. These patients’ data were not included in final data analysis.

### Scoring Criteria

The scoring of the DKQ involves summing up the scores of all the correct items of each participant, where higher score indicated better patient’s diabetes knowledge. One point was given to each correct answer and no point for the incorrect option.

### Study Participants and Setting

Inclusion criteria for the study were adult (>30 years) patient of type 2 DM, diagnosed at least 1 year before, with recent HbA1c lab test (not more than 8 weeks old form the date of interview), taking hypoglycemic medications and sufficient communication skills in the Urdu language. Patients with terminal illness or cognitive impairments and who could not complete the interviews were excluded. Study participants were interviewed face-to-face for collection of sociodemographic data and were assessed by using the translated (Urdu) version of DKQ. 130 eligible patients showed their willingness for participation in the study (response rate approximately 81.3%). Patient’s medical records were reviewed by the investigator on the same day for HbA1c levels, nature and number of hypoglycemic agents, and for the presence of comorbid conditions.

### Ethics Approval

This study was approved and carried out with the recommendations of the Monash University Human Research and Ethics Committee, Akuwat diabetic clinic Lahore, and Awan Medical Complex Lahore. Written consent was provided by all subjects before participation in the study, in accordance the Declaration of Helsinki.

### Statistical Analysis

The analyses were performed using SPSS 21.0.0 (SPSS Inc., Chicago, IL, USA). Normality of distribution of data was first determined by Kolmogorov–Smirnov test. Frequencies and descriptive statistics were used for demographics presentation, while means and SDs were calculated for the continuous variables. Scale items characteristics were evaluated by corrected item total correlations, corrected item sub-scale correlations, an increase of the scale’s reliability coefficient (Cronbach’s α) in case of item deletion and the items’ correlations with the HbA1c value. Cronbach’s α values were appraised by the following criteria: >0.9 = Excellent, >0.8 = Good, >0.70 = Acceptable, >0.6 = Questionable, >0.5 = Poor and <0.5 = Unacceptable (14).

One-way analysis of variance was conducted for known group’s validity, to compare the effect on patients’ DKQ scores, by HbA1c, after categorizing the respondents in to three groups on the basis of their HbA1c values. Patients with HbA1c values up to 7.5% were classified as “good glycemic control,” patients with values between 7.6 and 8.9% classified as “medium glycemic control,” and patients with values from 9.0% as “poor glycemic control.”

Multiple linear regression was calculated to predict patients’ DKQ sum score based on type of hypoglycemic agent, education, Hb1Ac, gender, BMI, age categories, and diabetes disease duration of the study participants. *P*-value of <0.05 (two-tailed test) was considered as criterion of statistical significance.

## Results

The study included 160 patients with type 2 diabetes in which 30 patients were excluded after data collection due to lack of either HbA1c results (*n* = 18) or insufficient information about their disease (*n* = 12). The mean age of the patients was 51.34 years (SD = 10.40), mean BMI was 29.68 (±6.16) kg/m^2^, with a slight preponderance of female gender (57.6%). Seventy-two (55.4%) were in age ranging 45–60 years and 25 (19.2%) were above 60 years of age. The majority of patients (38.5%) had no formal education, followed by university level (26.2%) and secondary level (25.4%) education. The mean duration of diabetes was 8.46 years (SD = 7.03). Majority of patients were on combination oral anti-diabetes medicines (42.3%) and did not use insulin for their diabetes management, followed by combination of oral hypoglycemic agents (OHA) and insulin (39.2%). Majority of the patients were using OHA alone (45%) or in combination with insulin (44%), whereas only 11% were using insulin exclusively. The mean duration of diabetes illness and HbA1c were 8.46 (±7.03) years and 8.55% (±1.91). Approximately 62% of the patients had HbA1c values above 7.5% (59.5 mmol/mol). The demographic characteristics of the patients are presented in Table 1, including the frequency distribution of the study patients and disease-related data.

**Table 1:** Demographic and disease characteristics of the study patients with differences in DKQ and HbA1c (*N* = 130).

Variables	Frequency (%)	DKQ Mean (SD)	*P*	HbA1c Mean (SD)	*P*
Age			0.52^a^		0.70^a^
<45 years	33 (25.4)	13.73 (±2.43)		8.46 (±1.85)	
45–60 years	72 (55.4)	13.08 (±2.95)		8.67 (±1.98)	
>60 years	25 (19.2)	13.20 (±2.34)		8.32 (±1.84)	
Gender					
Male	55 (42.3)	13.15 (±2.43)	0.11^b^	8.79 (±2.10)	0.18^b^
Female	75 (57.7)	13.36 (±2.91)		8.38 (±1.76)	
Education			0.04^a,^*		0.09^a^
No formal	50 (38.5)	13.23 (±3.27)		9.07 (±1.99)	
Primary	13 (10)	13 (±2.38)		8.39 (±1.59)	
Secondary	33 (25.4)	13.18 (±2.13)		8.31 (±1.93)	
University	34 (26.2)	14.32 (±2.14)		8.08 (±1.77)	
Diabetes duration			0.35^a^		0.30^a^
<5 years	47 (36.2)	12.96 (±2.91)		8.81 (±2.16)	
5–9 years	33 (25.4)	13.03 (±2.52)		8.36 (±1.71)	
10–14 years	30 (23.1)	13.40 (±2.76)		8.58 (±1.76)	
≥15 years	20 (15.4)	14.20 (±2.39)		7.99 (±1.77)	
Medication			0.14^a^		0.32^a^
Oral hypoglycemic agent (OHA) only	59 (45.4)	12.98 (±2.44)		8.81 (±2.07)	
Insulin only	14 (10.8)	12.43 (±2.06)		8.08 (±1.82)	
OHA + insulin	57 (43.8)	13.77 (±3.03)		8.39 (±1.74)	

*Data are M ± SD*.

*Significant (*P* < 0.05).

*Tests were ^a^one-way analysis of variance and ^b^independent sample t-test*.

*Coefficients that represent type 2 diabetes patients (*n* = 130) are DKQ, Diabetes Knowledge Questionnaire; HbA1c, glycated hemoglobin*.

### Internal Consistency

The items in this tool were found to be highly consistent internally. The Cronbach’s α for testing the internal consistency was 0.702 and in no case an item deletion led to a significant (*P* < 0.05) increase of the scale’s α coefficient (see Table 2). Two items (No. 3 and No. 15) have significant negative correlation with HbA1c, whereas item No. 17 and No. 20 sowed significantly (*P* < 0.05) positive correlation with HbA1c. Three items (No. 14, No. 18, and No. 23) showed highly significant (*P* < 0.01) positive correlation with HbA1c. A detailed overview of the above item characteristics is displayed in Table 2.

**Table 2:** Distribution of scores, test item difficulty (percent correct), discrimination (item-total correlation), internal consistency in case of deletion, and correlations with HbA1c of the 24-item Diabetes Knowledge Questionnaire at baseline (*N* = 130).

Item no.	Distribution of item scores	Percent correct	Corrected item-total item correlation	α if deleted	Correlation with HbA1c
1.	1.32 ± 0.54	26	0.151	0.70	−0.15
2.	1.34 ± 0.72	75	0.398	0.68	0.03
3.	2.02 ± 0.96	11	0.410	0.68	−0.20*
4.	2.14 ± 0.81	30	0.383	0.68	−0.06
5.	1.09 ± 0.39	95	0.289	0.69	−0.13
6.	1.34 ± 0.73	81	0.414	0.70	0.08
7.	1.27 ± 0.57	16	0.286	0.69	0.08
8.	1.07 ± 0.33	95	0.203	0.70	−0.08
9.	1.88 ± 0.58	60	0.298	0.67	0.01
10.	1.88 ± 0.44	75	0.190	0.66	−0.06
11.	1.56 ± 0.88	63	0.477	0.70	0.05
12.	2.03 ± 0.94	15	0.500	0.70	0.12
13.	1.35 ± 0.51	41	0.186	0.68	−0.07
14.	1.82 ± 0.83	45	0.345	0.70	0.24^†^
15.	1.06 ± 0.27	95	0.121	0.70	−0.18*
16.	1.05 ± 0.31	97	0.252	0.72	0.15
17.	1.31 ± 0.54	19	−0.164	0.73	0.18*
18.	1.42 ± 0.67	72	−0.194	0.69	0.24^†^
19.	1.22 ± 0.61	82	0.274	0.70	0.10
20.	1.33 ± 0.69	80	0.321	0.69	0.22*
21.	1.85 ± 0.71	47	0.229	0.70	−0.14
22.	1.92 ± 0.62	61	0.416	0.78	−0.05
23.	1.55 ± 0.67	35	−0.045	0.72	0.27^†^
24.	1.12 ± 0.35	12	0.077	0.70	−0.06

*Data are M ± SD, Pearson’s correlations, Cronbach’s α, or Spearman’s ρ*.

*Correlations with HbA1c are Spearman’s ρ*.

*P < 0.05 (two-tailed test).

*^†^P < 0.01 (two-tailed test)*.

### Patients’ Diabetes Knowledge

The median scores plus inter-quartile ranges (IQR) of DKQ and HbA1c with respect to patients’ demographics are presented in Table 1. Significant difference in DKQ scores was observed between educational levels of the patients. The DKQ with mean score 14.32 (±2.14) was observed with patients having university level education (*P* < 0.05), whereas no significant difference was observed in different age groups, gender, duration of diabetes, and insulin use (*P* > 0.05).

### Known Groups’ Validity

The mean HbA1c of the patients was 8.55% (±1.91). The results of one-way analysis of variance (ANOVA) revealed a statistically significant difference (*P* < 0.05) between DKQ scores of patients with “good glycemic control” (HbA1c ≤ 7.5%), “medium glycemic control” (HbA1c 7.6–8.9%), and “poor glycemic control” (HbA1c ≥ 9.0%) [*F*(2,127) = 5.336, *P* = 0.006]. *Post hoc* comparisons using Scheffe test indicated that the mean DKQ score of patients with “good glycemic control” (M = 14.22, SD 2.4) was statistically different from the patients with “poor control” (M = 12.56, SD 2.74). However, the mean DKQ score of patients with “medium glycemic control” (12.85 ± 2.74) did not statistically differ from the mean DKQ scores of patients with “good medium glycemic control” and “poor glycemic control,” detailed results are shown in Table 3.

**Table 3:** Comparison of the DKQ sum scores with patients’ HbA1c ≤ 7.5%, from 7.6 to 8.9%, and ≥9.0% (*N* = 130).

(I) Hb1Ac categories	Mean DKQ (±SD)	(J) Hb1Ac categories	Mean difference (I–J)	Sig.	95% Confidence interval	
					Lower bound	Upper bound
HbA1c ≤ 7.5% (*n* = 49)	14.22 ± 2.4	HbA1c 7.6–8.9%	1.37	0.066	−0.0708	2.8055
		HbA1c ≥ 9.0%	1.66*	0.010	0.3252	2.9933
HbA1c 7.6–8.9% (*n* = 35)	12.86 ± 2.74	HbA1c ≤ 7.5%	−1.37	0.066	−2.8055	0.0708
		HbA1c ≥ 9.0%	0.29	0.884	−1.1656	1.7495
HbA1c ≥ 9.0% (*n* = 46)	12.56 ± 2.75	HbA1c ≤ 7.5%	−1.66*	0.010	−2.9933	−0.3252
		HbA1c 7.6–8.9%	−0.29	0.884	−1.7495	1.1656

*Data are M ± SD*.

Tests were one-way ANOVA and Scheffe test for post hoc group comparisons. Scheffe test significance is expressed: *P < 0.05.

*DKQ, Diabetes Knowledge Questionnaire; HbA1c, glycated hemoglobin; ANOVA, analysis of variance*.

### Linear Logistic Regression

A significant regression equation was observed [*F*(7,119) = 2.472, *P* < 0.05], with an *R*^2^ of 0.076. Patients’ HbA1c (OR −0.17, CI −1.111 to 0.023) and education (OR 0.17, CI −0.032 to 0.758) were observed to be the significant predictors for DKQ sum score followed by longer diabetes disease duration (OR 0.13, CI −1.192 to 0.817) and older age (OR −0.11, CI −1.119 to 0.281). Whereas type of hypoglycemic medication, gender, and BMI categories were found to be least significant predicting variables for DKQ sum scores, details are presented in Table 4.

**Table 4:** Linear logistic regression between DKQ sum score and different variables of study participants (*N* = 130).

		95% CI	
Variable	OR	Lower bound	Upper bound
Type of hypoglycemic agent	0.09	−0.289	0.780
Education categories	0.17	−0.032	0.758
Hb1Ac categories	−0.17	−1.111	0.023
Gender	−0.06	−1.309	0.647
BMI categories	0.03	−0.517	0.755
Age categories	−0.11	−1.119	0.281
Diabetes disease duration	0.13	−0.192	0.817

*Coefficients that represent type 2 diabetes patients (*n* = 130) are DKQ, Diabetes Knowledge Questionnaire; HbA1c, glycated hemoglobin; BMI, body mass index. Type of hypoglycemic agent (ref OHA only); Education categories (ref No formal Education); HbA1c categories (ref ≤ 7.5%); gender (ref female); BMI categories (ref underweight); age categories (ref < 45 years); diabetes disease duration (ref < 5 years)*.

## Discussion

Optimum diabetes self-care is imperative for diabetes patients without adequate diabetes disease knowledge (9). Different questionnaire have been developed to examine and imparting diabetes disease knowledge. Without adequate disease knowledge, it seems quite difficult for diabetic patients to practice adequate self-care activities. Most of the available questionnaires have multiple options or too lengthy, which makes these difficult to administer to the diabetes patients in developing countries with low literacy rate (9, 15, 16). Moreover, very few are valid and reliable, and none is available in Urdu language. DKQ is a relatively simple study tool with three options (Yes, No, or don’t know) to select and covering major domains for disease knowledge assessment, like diet, blood glucose self-monitoring, physical activity, and medication intake behavior. A critical review of the patient’s response can be used to educate patients and provide opportunities for further research in this domain. Thus, the main objective of this study was to translate the English version of DKQ in to Urdu language and second evaluate the psychometric properties of the translated Urdu version of the DKQ using a convenience sample of patients with type 2 diabetes in Pakistan. This study was the first to systematically translate and validate the 24-item DKQ in Urdu language.

Overall, a good internal consistency (Cronbach’s α) was observed for the Urdu version of DKQ in our study among Pakistani type 2 diabetes, which is comparable to its Spanish version (10).

Many studies report a significant association of glycemic control with patients’ diabetes disease knowledge (17–19). In this study, patients’ glycemic control was assessed by their recent HbA1c test, and patients with good glycemic control (HbA1c ≤ 7.5%) scored significantly higher (*P* < 0.05) DKQ scores as compared to patients with HbA1c values greater than 7.5%, this finding is in line with that of Al-Qazaz study (19).

It was revealed by multiple linear regression analysis that patients’ education, age, HbA1c level, and diabetes disease duration are important predictors for DKQ sum scores. Better disease knowledge was observed in patients with longer disease duration and older age, whereas gender, body mass index, and type of hypoglycemic agents were insignificantly associated with patients’ DKA score. Results of this study indicate that lower education level has an impact on diabetes knowledge, and it is similar to the evidence shown in earlier studies (20, 21). So, patients with higher education and longer disease duration were observed to have better diabetes disease knowledge and better glycemic control too.

## Conclusion

There is need to assess and improve the patients’ diabetes knowledge, which is very important for an effective self-care and achieving optimum glycemic control. The findings of this study support that the Urdu version of the DKQ a valid instrument for measuring patients’ diabetes knowledge. This finding is in line with our hypotheses as patients with higher DKQ scores were expected to have better glycemic control. The good associations between glycemic control and scores of the Urdu version of DKQ suggest that helping patients to improve their diabetes disease knowledge might lead to improved self-care and reduced risks of disease-related complications.

### Strength and Limitations

This study is the first to translate and validate the DKQ in Urdu language. A possible limitation of this study is that it was used to assess diabetes knowledge in type 2 diabetes patients; diabetes type 1 patients should also be recruited to establish validity and reliability of this tool across both major diabetes types. Low literacy rate of the respondents is another limitation of this study, as it might limit generalizability to other patient groups. However, the results of this study could be generalized to the community as the data were collected from the institutional diabetic clinics, which cater the needs of majority of diabetic patients. The strength of this study lies in the standardized data assessment using structured interviews and HbA1c analysis in one laboratory. Moreover, DKQ is relatively easy to administer to administer.

## Ethics Statement

This study was approved and carried out with the recommendations of the Monash University Human Research and Ethics Committee (MUHREC), Akuwat diabetic clinic Lahore, and Awan Medical Complex Lahore. Written consent was provided by all subjects before participation in the study, in accordance with the Declaration of Helsinki.

## Author Contributions

AB and TK: conception, design of the work, the acquisition, analysis, interpretation of data for the work, drafting the work, revising it critically, and final approval of the version to be published. PP, SL, and AK: design of the work, interpretation of data for the work, drafting the work, revising it critically, and final approval of the version to be published. AB, TK, PP, AK, and SL agreed to be accountable for the content of the work.

## Conflict of Interest Statement

The authors declare that the research was conducted in the absence of any commercial or financial relationships that could be construed as a potential conflict of interest.

## Appendix

**Table A1:** Diabetes Knowledge Questionnaire (Urdu Version).

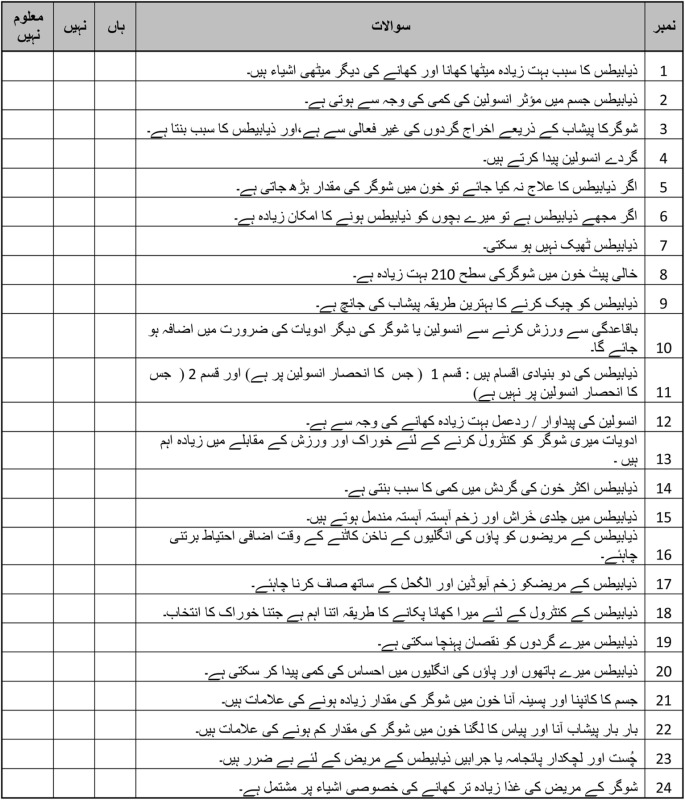

*Translated and Adapted by Allah Bukhsh and Tahir Mehmood Khan (allah.bukhsh@monash.edu, tahir.mehmood@monash.edu) School of Pharmacy, MONASH University, Malaysia, 2016*.

*This product was adapted from the DKQ “Diabetes Knowledge Questionnaire”—Garcia and Associates for the diabetes self-management project at Gateway Community Health Center, Inc. with support from the Robert Wood Johnson Foundation^®^ in Princeton, NJ, USA*.
